# Clinical and Functional Results of Reverse Total Shoulder Arthroplasty and Postoperative Rehabilitation Protocol

**DOI:** 10.7759/cureus.23322

**Published:** 2022-03-19

**Authors:** Vitor C Pereira, José Barreto, Sónia Tomé, João Cunha, João Amaro, Jorge Moreira, António Miranda, Catarina A Branco

**Affiliations:** 1 Department of Physical Medicine and Rehabilitation, Centro Hospitalar de Entre o Douro e Vouga, Santa Maria da Feira, PRT; 2 Department of Physical Medicine and Rehabilitation, Hospital da Senhora da Oliveira Guimarães, Guimarães, PRT; 3 Department of Occupational Health, Centro Hospitalar Universitário de São João, Porto, PRT; 4 Department of Orthopaedics and Traumatology, Centro Hospitalar de Entre o Douro e Vouga, Santa Maria da Feira, PRT

**Keywords:** rehabilitation, shoulder rehabilitation, postoperative treatment, rotator cuff tear arthropathy, reverse total shoulder arthroplasty

## Abstract

Purpose

This retrospective study aims to analyze the clinical and functional results obtained over a seven-year period of performing reverse total shoulder arthroplasty (RTSA) and the subsequent postoperative rehabilitation protocol.

Methods

We analyzed data from 80 patients who were evaluated at a preoperative, as well as monthly postoperative outpatient consultation, until the discharge from the rehabilitation program, using Constant Score (CS).

Results

A comparison of preoperative and postoperative (after rehabilitation protocol) results revealed an improved functional score of absolute CS (20.8 increase), normal relative CS (29.1 increase), and individual relative CS (31.7 increase) with statistical significance (p<0.05). From the analysis of CS subscores, there was a positive evolution of the pain subscore, as well as flexion, abduction, and external rotation combined with abduction range of motion (ROM). Contrarily, there was a negative evolution of the combined internal rotation, extension, and adduction ROM, as well as deltoid muscle strength. No statistically significant correlations were found between age and postoperative CS, as well as between the time interval from surgery to the beginning of outpatient rehabilitation and CS evolution.

Conclusion

Our study demonstrates that RTSA is an effective therapeutic option that, if combined with a well-structured rehabilitation program, can improve pain, mobility, and upper limb functionality.

## Introduction

Shoulder pathology is common worldwide, representing a growing concern for the elderly, workers, and athletes [1]. Reverse total shoulder arthroplasty (RTSA) is a valid and widespread therapeutic option in cases of glenohumeral arthropathy resistant to conservative treatment [2-5]. Initially, it has been designed as an alternative to the conventional total arthroplasty and hemiarthroplasty in cases of shoulder arthropathy associated with extensive rotator cuff tears [6]. Currently, taking into account the good results obtained, it is also indicated for rheumatoid arthritis, proximal humeral fractures, humeral head osteonecrosis, and revision of total shoulder arthroplasty [2-7].

From a biomechanical point of view, in RTSA, the center of rotation moves medially and inferiorly, increasing both the deltoid muscle lever arm and the recruitment of its anterior and posterior fibers [8]. These changes facilitate shoulder elevation movement, with greater amplitude until a subacromial conflict occurs [2]. Several factors influence the functional results of RTSA, such as surgical indication, surgeon's experience, surgical technique, patient characteristics, and postoperative rehabilitation [9].

In our hospital, RTSA is considered in the diagnoses described above when shoulder pain persists and upper limb functionality is compromised despite conservative treatment. For this purpose, clinical assessment through Constant Score (CS) proves to be very useful. CS is a validated and consensual functional shoulder assessment tool and is recommended by the European Society for Surgery of the Shoulder and the Elbow and British Elbow and Shoulder Society [10]. Regarding the surgical technique, RTSA in our institution is performed through a deltopectoral approach with biceps brachii long portion tenotomy with subsequent tenodesis if possible. Usually, the subscapularis is preserved (avoiding its tenotomy) and no tendon transfers from the large dorsal and major round are performed.

Given the increasing use of RTSA at our hospital, the Physical Medicine and Rehabilitation (PMR) and Orthopedics departments have jointly developed a rehabilitation protocol [2] aimed to standardize and improve the approach of these patients. Based on the available scientific evidence, the protocol has been designed to provide rehabilitation care in order to optimize the functional recovery of postoperative patients. Three key points of orientation for the postoperative rehabilitation of this arthroplasty are common, namely, joint protection, optimization of deltoid muscle function, and delineation of functional expectations [2,11]. These guidelines are common throughout the three phases of the protocol (Appendices). In the first phase (day one to week six), the patient must perform the immobilization of the sling-operated limb for four weeks. During this phase, passive mobilization until 90 degrees of shoulder elevation in the plane of the scapula, Codman's pendular exercises, and deltoid muscle strengthening (through neuromuscular electrical stimulation and isometric muscle strengthening exercises) should begin. After immobilization is removed, active mobilization of the operated shoulder is recommended, maintaining restriction of combined extension, internal rotation, and adduction movement during the first 12 postoperative weeks. In this second phase (week six to 12), progresses in the deltoid muscle strengthening through dynamic exercises should occur, and hydrokinesitherapy may also be useful. In a third phase (from week 12), the objectives should focus on the progression of strengthening and the promotion of functional independence in activities of daily living.

The aim of this study is to analyze the clinical and functional results obtained in a seven-year period of application of RTSA and the subsequent postoperative rehabilitation protocol developed in our hospital.

## Materials and methods

This retrospective study included 80 patients who underwent RTSA surgery with consequent integration in the RTSA rehabilitation protocol at Centro Hospitalar de Entre o Douro e Vouga, from August 1, 2011, to November 31, 2018 [2]. We consulted and analyzed data from the functional assessment through the CS at a preoperative, as well as monthly postoperative outpatient consultation until the discharge from the rehabilitation program.

Both the rehabilitation protocol and the data collected from the functional assessment through the CS are performed as usual clinical practice at our institution. Therefore, patients are evaluated preoperatively with CS (except in cases of humeral fracture) in order to determine the previous functional state of the shoulder joint, as well as transmit and manage expectations of recovery to the patient. In the early days of the postoperative phase, still in the orthopedics hospitalization, patients are evaluated by a PMR physician for their inclusion in the rehabilitation program. This visit allows the teaching of recommended postoperative care, such as the avoidance of movements that cause prosthesis dislocation and Codman pendulum exercises. It also enables the prescription of rehabilitation treatment (according to the rehabilitation protocol [2]), to start on an outpatient basis at our PMR department after hospital discharge. Subsequently, postoperative serial evaluations are performed at the PMR outpatient visits in order to adapt the therapeutic prescription to the clinical and functional evolution of the patient. In each of these evaluations, which occur monthly until the date of discharge from the rehabilitation program, functional evaluation is again performed through the CS. Of note, the range of motion measurements included in the CS are made using a manual goniometer. Discharge from the rehabilitation program is considered when both a clinical and a functional plateau of improvement is achieved, after a favorable evolution period. In addition to calculating the absolute CS from the different subscores, the normal relative CS and the individual relative CS are also calculated. The normal relative CS corresponds to the ratio between the affected shoulder CS and the standardized CS for the age and gender of the healthy population. The individual relative CS concerns the quotient between the affected shoulder and the contralateral shoulder of the patient.

Statistical analysis of the data was performed using the Statistical Package for Social Sciences (SPSS) software version 20.0 (2012, Chicago, IL, USA). For the comparison of preoperative and postoperative CS values, as well as for its different subscores, we used the paired sample t-test. To analyze the correlations between age and postoperative CS, as well as the time interval from surgery to the beginning of the outpatient rehabilitation program and the CS evolution, Pearson’s correlation coefficient was used. A 5% significance level was adopted in all statistical tests. Thus, statistically significant associations were considered when the p-value was less than 0.05.

All authors reviewed and approved the final manuscript. This study was also reviewed by our institutional ethics committee, which waived the need for approval and informed consent due to the retrospective nature of the investigation.

## Results

Between August 1, 2012, and August 1, 2019, 80 patients undergoing RTSA at our hospital were included in the study, with a mean age of 69.2 years. Of these, 55 (68.8%) were female and 25 (31.2%) were male. Forty-eight (48; 60%) patients underwent right shoulder RTSA and 32 (40%) underwent left shoulder surgery. No patient in this study underwent surgery on both shoulders during the follow-up period, although 23 patients (28.8%) had contralateral shoulder pathology (Table 1).

**Table 1:** Sample characterization

Sample characterization	Reverse total shoulder arthroplasty (n=80)		
	Mean or n		Standard deviation or %
Age (years)	69.2		8.4
Gender	Female	55	68.8%
	Male	25	31.2%
Upper limb undergoing surgery	Right	48	60.0%
	Left	32	40.0%
Contralateral upper limb pathology	Yes	23	28.8%
	No	57	71.2%
Surgery indication	Rotator Cuff Arthropathy	52	65.8%
	Proximal Humeral Fractures	21	26.6%
	Rheumatoid Arthritis	4	5.1%
	Arthroplasty Revision	2	2.5%
Time until the start of the outpatient rehabilitation program (weeks)	3.6		2.8
Rehabilitation program duration (weeks)	18.2		8.6

The preoperative diagnosis was glenohumeral osteoarthritis secondary to extensive rotator cuff tear in 52 patients (65.8%), proximal humeral fracture in 21 (26.6%), glenohumeral arthropathy secondary to rheumatoid arthritis in 4 (5.1%) and revision of arthroplasty in two patients (2.5%) (Table 1).

The mean time from surgery until the start of the outpatient rehabilitation program at the PMR department was approximately 25 (± 11) days. The mean duration of the rehabilitation program was 139 (±56) days. Analyzing according to the preoperative diagnosis, there was a higher mean duration of the rehabilitation program in patients with a humeral fracture (186 days) as compared to the other groups (124 days), with statistical significance in the test (p <0.05).

Regarding the preoperative evaluations of the CS, a mean of absolute CS of 36.9 (± 13.3), normal relative CS of 52.0% (± 19.5), and individual relative CS of 52.4% (± 19.7) were observed (Table 2). At the time of discharge from the rehabilitation program, a mean of absolute CS of 57.7 (± 9.0), a normal relative CS of 81.1% (± 15.3), and an individual relative CS of 84.1% (± 23.3) were obtained. A comparison of pre and postoperative results revealed variations with statistical significance of absolute SC (20.8 increase), normal relative SC (29.1 increase), and individual relative SC (31.7 increase) (p <0.05).

**Table 2:** Preoperative and postoperative (at the date of discharge from the rehabilitation program) Constant Score values M = mean, Sd = Standard deviation

Constant Score Values	Preoperative M (Sd)	Postoperative M (Sd)
Absolute	36.9 (13.3)	57.7 (9.0)
Normal Relative	52.0% (19.5)	81.1% (15.3)
Individual Relative	52.4% (19.7)	84.1% (23.3)

The different subscores that compose the CS were also analyzed regarding preoperative and postoperative means (Table 3). In respect of the pain subscore, the preoperative mean was 4.2 (± 4.5) while the postoperative mean was 13.7 (± 2.2). Concerning the level of hand activity, a preoperative mean of 9.0 (± 3.1) and a postoperative mean of 17.0 (± 4.5) were observed. Flexion, abduction, external rotation combined with abduction, and internal rotation combined with adduction movements were also evaluated. The preoperative means were 4.1 (± 2.3), 3.6 (± 2.0), and 4.7 (± 3.3), 4.1 (± 1.5), respectively, while the postoperative means were 6.6 (± 1.6), 5.5 ( ± 1.6), 6.7 (± 1.9), 3.6 (± 2.0), respectively. Regarding the muscle strength subscore, a preoperative mean of 7.7 (± 4.1) and a postoperative mean of 4.6 (± 1.3) were obtained.

**Table 3:** Preoperative and postoperative values of different subscores of Constant Score (paired sample t-test) *Statistically significant CS - Constant Score. Preop - Preoperative. Postop - Postoperative. Sd - Standard Deviation.

CS subscores	Preop Mean (Sd)	Postop Mean (Sd)	Pre-Postop Difference - Mean	P-value
A. Pain	4.2 (4.5)	13.7 (2.2)	9.5	<0.001^*^
B. Level of Hand Activity	9.0 (3.1)	17.0 (4.5)	8.0	<0.001^*^
C. Mobility	16.4 (7.9)	22.5 (5.0)	6.1	<0.001^*^
C1. Flexion	4.1 (2.3)	6.6 (1.6)	2.5	0.003^*^
C2. Abduction	3.6 (2.0)	5.5 (1.6)	1.9	0.001^*^
C3. External Rotation + Abduction	4.7 (3.3)	6.7 (1.9)	2.0	<0.001^*^
C4. Internal Rotation + Adduction + Extension	4.1 (1.5)	3.6 (2.0)	-0.5	0.596
D. Muscle strength	7.7 (4.1)	4.6 (1.3)	-3.1	0.999

From the analysis of these results, there was a positive and statistically significant evolution (p <0.05) of pain subscore, as well as flexion, abduction, and external rotation combined with abduction range of motion. On the other hand, a negative evolution of the deltoid muscle strength and the combined internal rotation, extension, and adduction range of motion were observed. However, these latter results were not statistically significant (Table 3).

No statistically significant differences were found regarding the correlation between age and postoperative CS. In addition, there was no statistically significant difference between the time interval from surgery to the beginning of the outpatient rehabilitation program (less than or greater than 20 days) and the CS evolution.

## Discussion

From the analysis of the results of the application of the RTSA protocol, the favorable functional evolution of the patients after its application is highlighted. This can be inferred from the statistically significant increase, at the time of discharge from the rehabilitation program, of the postoperative CS values compared to the preoperative values. In fact, although this prosthesis was initially used in elderly patients (aged 76-89 years), a growing number of publications describe its use in considerably younger patients [12-15]. This is largely due to the good results obtained, as well as their reproducibility.

From the evaluation of the CS subscores, the pain subscore stands out as the one with the highest evolution, followed by the level of hand activity. In terms of mobility, we found a statistically significant improvement in flexion, abduction, and abduction combined with external rotation range of motion. These results are in agreement with the existing literature, which shows an improvement in pain, as well as a progression of flexion and abduction mobility after RTSA, due to the increase of the lever arm and consequent optimization of deltoid muscle function [12]. However, the literature is inconsistent concerning the postoperative improvement of external rotation motion [9,15-16]. The authors consider that this difference may be due to the type of prosthesis and surgical technique used, to the absence of an isolated evaluation of external rotation movement in the CS, but especially to the preoperative state of these muscles. Besides being reported that the posterior deltoid activates the external rotation movement with the abducting shoulder [17-18], it is known that the degree of internal and external rotation achieved in the postoperative period is largely influenced by the preoperative state of the rotator muscles [19-20]. In addition, lateralized prostheses appear to provide a more sustained improvement in external rotational range of motion compared to medialized prostheses [18,21]. In order to obtain better clinical and functional results in terms of external rotation, new surgical techniques are being performed, namely, the latissimus dorsi and teres major tendon transfers [9,11,22]. Patients undergoing this procedure have experienced an improvement in pain and functionality, but a higher rate of complications may be associated. Lewis L. Shi et al. recommend that this technique be limited to patients with an indication for RTSA, associated with marked external rotation deficit and a high degree of infraspinatus/teres minor fat infiltration [23]. In our sample, none of the patients underwent tendon transfer.

According to the analysis of the existing literature, there is a lower magnitude evolution than the results described in this article [7,24-26]. That can be explained by different follow-up times, so the authors consider that the follow-up time of these patients should be extended in order to assess the long-term evolution of the obtained results. According to Uschock et al., rehabilitation seems to improve several objective and subjective aspects of shoulder function, both in the medium and long term. This applies to the range of motion and functionality in daily living activities but seems to have no effect on the residual pain level and muscle strength of the affected shoulder [27]. For more effective pain control, the use of cryotherapy and analgesic electrotherapy (both prescribed to our patients) as well as radiofrequency and massage should be considered.

With regard to the negative evolution of the internal rotation, extension, and adduction combined movement, this can be interpreted by restrictions inherent to the biomechanics of these prostheses and the fact that it constitutes its luxant movement [19,28]. This position allows the prosthesis "to escape” anteriorly and inferiorly, constituting the most vulnerable position of the prosthesis [11]. Therefore, it was avoided during the first 12 weeks postoperatively.

Concerning the strength subscore, negative evolution is not completely defined in the literature [24]. However, according to Roy et al., strength recovery for gender and age normal values is not expected postoperatively [29]. According to the same author, there is a low agreement between strength and functionality, and apparently, patients with low muscle strength may have good functionality after surgery [29]. Other authors found different results in the evaluation of this same correlation. According to Bergmann et al., the presence of a passive range of motion greater than the active one can be interpreted by decreasing the torque generated in the joint, rather than by the structural limitation caused by the prosthesis [30]. In addition to the foregoing, we consider that this negative evolution may still be due to the following factors: interobserver variability (as preoperative and postoperative assessments are performed by different physicians), to the muscular atrophy itself due to initial postoperative immobilization, and limitations both in terms of load and upper limb function resulting from this arthroplasty. In this sense, the need to extend the rehabilitation time to enhance deltoid muscle strengthening should be considered. Another strategy that could be implemented in order to improve this outcome would be the inclusion of these patients (except in the case of fracture) in a preoperative rehabilitation program.

From the analyzed scientific literature, we did not find references to the mean times of RTSA rehabilitation programs according to the preoperative pathology. Nevertheless, in this study, we found a longer rehabilitation time for fractures. This find may be related to the longer immobilization time to which these patients are subjected, with the consequent delay in the rehabilitation program progression.

Study limitations

This study has some limitations, namely, its retrospective nature, which affects its reliability. Additionally, the non-use of absolute values in the range of motion evaluation may have implications on the evolution of scores since the presence of wide ranges may condition lower responsiveness. Compared to the studies evaluated, in the present study no instruments of satisfaction, quality of life, or participation were used, which limits the conclusions to be drawn. According to Samitier et al., RTSA is a surgical procedure with high patient satisfaction, regardless of the type of prosthesis or surgical indication [21]. Finally, the short follow-up time limits the ability to detect potential complications or long-term functionality evolution, so we believe that further and larger studies will be needed.

## Conclusions

RTSA is an increasingly common therapeutic option in cases of glenohumeral arthropathy resistant to conservative treatment, and its indications have increased over time. Given the increasing use of this surgical approach at our hospital, the Physical Medicine and Rehabilitation and Orthopedics departments aimed to standardize and improve the approach of these patients. Our study demonstrates that RTSA is an effective therapeutic option that, when combined with a well-structured rehabilitation program, can improve pain, mobility, and upper limb functionality.

## Appendices

**Table 4:** Reverse total shoulder arthroplasty (RTSA) rehabilitation protocol developed by the Physical Medicine and Rehabilitation and Orthopedics departments of Centro Hospitalar de Entre o Douro e Vouga

Phase I (Day 1 – week 6)	Goals	Control pain and inflammation, promote healing of soft tissue, and maintain the integrity of the replaced joint Restore active range of motion (ROM) of the elbow, wrist, and hand
	Precautions	- Keep the incision clean and dry - Sling is worn for 4 weeks (6 weeks if RTSA procedure is a revision surgery) postoperatively and only removed for rehabilitation and bathing.
	Interventions	Cryotherapy (4 times a day for about 15 minutes); Analgesic electrotherapy; Active-Assisted ROM of the elbow, wrist, and hand; Progressive passive shoulder ROM: forward flexion and elevation in the scapular plane in supine to 120 degrees; Abduction to 45 degrees; External rotation to 20-30 degrees; No extension, adduction, and internal rotation; Neuromuscular electrical stimulation of deltoid muscle; Sub-maximal pain-free deltoid and periscapular isometrics in the scapular plane; Gentle resisted exercise of the elbow, wrist, and hand.
Phase II (week 6-12)	Goals	Gradually restore shoulder active ROM. Allow continued healing of soft tissue
	Precautions	Do not lift heavy objects; No supporting of body weight by the involved upper extremity
	Interventions	Cryotherapy, analgesic electrotherapy, and neuromuscular electrical stimulation as needed; Begin shoulder active-assisted/active ROM in supine with progression to sitting/standing: forward flexion and elevation in the scapular plane as tolerated, external rotation, internal rotation (to 50 degrees); Begin gentle periscapular and deltoid sub-maximal pain-free isotonic strengthening exercises; Begin gentle glenohumeral internal rotators and external rotators sub-maximal pain-free isometrics. Progress strengthening of the elbow, wrist, and hand; Individualized hydrokinesitherapy; Occupational therapy: begin functional activities and activities of daily living with involved upper extremity
Phase III (> 12 weeks)	Goals	Enhance functional use of operative extremity; Achieve independence in activities of daily living
	Precautions	No sudden lifting or pushing activities.
	Interventions	Continue with the previous rehabilitation program as needed; Progressing in shoulder active ROM in standing position; Progressing in periscapular, deltoid, and shoulder rotators strengthening exercises; Occupational therapy: progressing in functional activities and promoting independence in activities of daily living

## References

[1] Killian ML, Cavinatto L, Galatz LM, Thomopoulos S (2012). Recent advances in shoulder research. Arthritis Res Ther.

[2] Amaro J MJ, Miranda A, Aguiar-Branco C (2012). Reabilitação da Artroplastia do Ombro com Prótese Total Invertida [Article in Portuguese]. Revista da SPMFR.

[3] Hatzidakis A NT, Boileau P (2005). Reverse shoulder arthroplasty indications, technique, and results. Tech Surg Shoulder Elbow Surg.

[4] Walch G, Boileau P, Noël E (2010). Shoulder arthroplasty: evolving techniques and indications. Joint Bone Spine.

[5] Mengshoel AM, Slungaard B (2005). Effects of shoulder arthroplasty and exercise in patients with rheumatoid arthritis. Clin Rheumatol.

[6] Mole D, Favard L (2007). Excentered scapulohumeral osteoarthritis [Article in French]. Rev Chir Orthop Reparatrice Appar Mot.

[7] Rittmeister M, Kerschbaumer F (2001). Grammont reverse total shoulder arthroplasty in patients with rheumatoid arthritis and nonreconstructible rotator cuff lesions. J Shoulder Elbow Surg.

[8] Wilcox RB, Arslanian LE, Millett P (2005). Rehabilitation following total shoulder arthroplasty. J Orthop Sports Phys Ther.

[9] França FO, Freitas JM, Godinho PC, Gonçalves DM, Vieira T, Pereira US (2018). Clinical and functional evaluation of patients submitted to reverse arthroplasty with minimum one year of follow-up. Rev Bras Ortop.

[10] Walton MJ, Walton JC, Honorez LA, Harding VF, Wallace WA (2007). A comparison of methods for shoulder strength assessment and analysis of Constant score change in patients aged over fifty years in the United Kingdom. J Shoulder Elbow Surg.

[11] Boudreau S, Boudreau ED, Higgins LD, Wilcox RB 3rd (2007). Rehabilitation following reverse total shoulder arthroplasty. J Orthop Sports Phys Ther.

[12] Alcobia-Diaz B, Lopiz Y, Garcia-Fernandez C, Rizo de Alvaro B, Marco F (2017). Patient reported activities after reverse total shoulder arthroplasty in rotator cuff arthropathy patients. Rev Esp Cir Ortop Traumatol.

[13] Galasso O, Familiari F, Gasparini G (2015). Treatment options for irreparable postero-superior cuff tears in young patients. World J Orthop.

[14] Gerber C, Wirth SH, Farshad M (2011). Treatment options for massive rotator cuff tears. J Shoulder Elbow Surg.

[15] Muh SJ, Streit JJ, Wanner JP (2013). Early follow-up of reverse total shoulder arthroplasty in patients sixty years of age or younger. J Bone Joint Surg Am.

[16] Lädermann A, Lo EY, Schwitzguébel AJ, Yates E (2016). Subscapularis and deltoid preserving anterior approach for reverse shoulder arthroplasty. Orthop Traumatol Surg Res.

[17] Ackland DC, Richardson M, Pandy MG (2012). Axial rotation moment arms of the shoulder musculature after reverse total shoulder arthroplasty. J Bone Joint Surg Am.

[18] Wolff AL, Rosenzweig L (2017). Anatomical and biomechanical framework for shoulder arthroplasty rehabilitation. J Hand Ther.

[19] Boileau P, Watkinson D, Hatzidakis AM, Hovorka I (2006). Neer Award 2005: the Grammont reverse shoulder prosthesis: results in cuff tear arthritis, fracture sequelae, and revision arthroplasty. J Shoulder Elbow Surg.

[20] Karelse AT, Bhatia DN, De Wilde LF (2008). Prosthetic component relationship of the reverse Delta III total shoulder prosthesis in the transverse plane of the body. J Shoulder Elbow Surg.

[21] Samitier G, Alentorn-Geli E, Torrens C, Wright TW (2015). Reverse shoulder arthroplasty. Part 1: systematic review of clinical and functional outcomes. Int J Shoulder Surg.

[22] Boileau P, Watkinson DJ, Hatzidakis AM, Balg F (2005). Grammont reverse prosthesis: design, rationale, and biomechanics. J Shoulder Elbow Surg.

[23] Shi LL, Cahill KE, Ek ET, Tompson JD, Higgins LD, Warner JJ (2015). Latissimus dorsi and teres major transfer with reverse shoulder arthroplasty restores active motion and reduces pain for posterosuperior cuff dysfunction. Clin Orthop Relat Res.

[24] Alta TD, Veeger HE, Janssen TW, Willems WJ (2012). Are shoulders with a reverse shoulder prosthesis strong enough? A pilot study. Clin Orthop Relat Res.

[25] Favard L, Levigne C, Nerot C, Gerber C, De Wilde L, Mole D (2011). Reverse prostheses in arthropathies with cuff tear: are survivorship and function maintained over time?. Clin Orthop Relat Res.

[26] Valenti P, Sauzières P, Katz D, Kalouche I, Kilinc AS (2011). Do less medialized reverse shoulder prostheses increase motion and reduce notching?. Clin Orthop Relat Res.

[27] Uschok S, Herrmann S, Pauly S, Perka C, Greiner S (2018). Reverse shoulder arthroplasty: the role of physical therapy on the clinical outcome in the mid-term to long-term follow-up. Arch Orthop Trauma Surg.

[28] Smith CD, Guyver P, Bunker TD (2012). Indications for reverse shoulder replacement: a systematic review. J Bone Joint Surg Br.

[29] Roy JS, Macdermid JC, Goel D, Faber KJ, Athwal GS, Drosdowech DS (2010). What is a successful outcome following reverse total shoulder arthroplasty?. Open Orthop J.

[30] Bergmann JH, de Leeuw M, Janssen TW, Veeger DH, Willems WJ (2008). Contribution of the reverse endoprosthesis to glenohumeral kinematics. Clin Orthop Relat Res.

